# Improving our understanding of the *in vivo* modelling of psychotic disorders: A protocol for a systematic review and meta‐analysis

**DOI:** 10.1002/ebm2.22

**Published:** 2017-03-17

**Authors:** Zsanett Bahor, Cristina Nunes‐Fonseca, Lindsay D.G. Thomson, Emily S. Sena, Malcolm R. Macleod

**Affiliations:** ^1^ Centre for Clinical Brain SciencesUniversity of EdinburghEdinburghUK; ^2^ Division of PsychiatryUniversity of Edinburgh, Royal Edinburgh HospitalEdinburghUK

**Keywords:** psychosis, animal models, systematic review, meta‐analysis, protocol

## Abstract

Psychosis represents a set of symptoms against which current available treatments are not universally effective and are often accompanied by adverse side effects. Clinical management could potentially be improved with a greater understanding of the underlying biology and subsequently with the introduction of novel treatments. Since many clinical drug candidates are identified through *in vivo* modelling, a deeper understanding of the pre‐clinical field, might help us understand why translation of results from animal models to inform mental health clinical practice has so far been weak. We set out to give a shallow, but broad unbiased overview of experiments looking at the *in vivo* modelling of psychotic disorders using a systematic review and meta‐analysis. This protocol describes the exact methodology we propose to follow in order to quantitatively review both studies characterizing a model and those experiments that investigate the effects of novel therapeutic options. We are interested in assessing the prevalence of the reporting of measures to reduce risk of bias, and the internal and external validity of the animal models and outcome measures used to validate these models. This generation of strong empirical evidence has the potential to identify areas for improvement, make suggestions for future research avenues, and ultimately inform what we think we know to improve the current attrition rate between bench and bedside in psychosis research. A review like this will also support the reduction of animal numbers used in research and the refinement of experiments to maximize their value in informing the field.

## 1 GENERAL INFORMATION

We have used the proposed framework of Systematic Review Protocol for Animal Intervention Studies for the construction of this protocol.^1^

### 1.1 Title of the review

Improving our understanding of the *in vivo* modelling of psychotic disorders.

### 1.2 Stage of review at time of protocol

**Table 1:** Stage of review at time of protocol registration

Review stage	Started	Completed
Preliminary searches	Yes	Yes
Piloting of the study selection process	Yes	Yes
Formal screening of search results against eligibility criteria	Yes	Yes
Data extraction	Yes	No
Reporting of risk of bias (quality) assessment	Yes	No
Data analysis	No	No

## 2 OBJECTIVES

### 2.1 Background

A mental disorder is defined as “any disorder or disability of the mind” by The Mental Health Act 2007 for England and Wales^2^ and as “mental illness, learning disability or personality disorder however caused or manifested” by the Mental Health (Care and Treatment) (Scotland) Act 2003.^3^ The definition in legal terms is therefore very wide.

Clinically, this is also the case when considering international classifications of mental disorders.^4^, ^5^ Results from scientific literature databases such as PubMed reveal that mental disorders at the cognitive–emotional interface have received considerable and increasing attention in the field of research over the last decade. Psychotic mental disorders represent a severe category of these mental disorders that, in England, have an overall incidence rate of about 32 per 100 000 person‐years.^6^

These disorders are characterized by disorganized thinking and perceptions that manifest themselves most commonly through delusions and hallucinations. Other symptoms include abnormal motor behaviour and negative symptoms such as apathy and blunted affect (lack of emotional response), mostly associated with schizophrenia and less so with other psychotic disorders.^4^, ^7^ Broadly speaking, we can group psychotic disorders into 4 main diagnostic categories, which include: (1) non‐affective psychotic disorders or disorders that are primarily considered to be psychotic disorders and include examples like schizophrenia and schizoaffective disorder; (2) affective psychotic disorders, or mood disorders, that present with psychotic features, like bipolar disorder or major depressive disorder with psychotic features; (3) substance‐induced psychotic disorders that can be any substance‐ or medication‐induced psychosis; and (4) psychotic disorders due to a general medical condition.^4^ Incidence rates among the English population are the highest for non‐affective psychoses (23 per 100 000 person‐years), with schizophrenia accounting for about 15 per 100 000 person‐years.^6^

Pharmacological treatment remains the cornerstone of the clinical management of these disorders. These treatments were introduced in the 1950s and a second generation of drugs in the 1990s, but whilst efficacious, they regrettably remain problematic in terms of side effects and treatment resistance. Adverse side effects accompany many anti‐psychotic medications, and partial or intermittent symptomatic relief is common, contributing to non‐compliance.^8^, ^9^ Most concerning, however, is the group of individuals experiencing psychosis who are treatment‐resistant.^10^ Our understanding of the underlying mechanisms for these disorders and how to ameliorate them remains poor, leading to a struggle in the development of newer and better pharmacological treatment options.^11^ Despite the large amount of preclinical research in the field attempting to advance our understanding of these disorders, development of novel drug classes for psychiatric diseases is still an area of current weakness, and no real breakthroughs have been made over the last decade.^8^

This shortcoming in translation of promising interventions from bench to bedside could potentially be attributed to methodological flaws in preclinical experiments or the inadequacy of the animal models themselves when it comes to representing the human condition.^12^ Modelling disorders in animals is especially difficult in neuropsychiatry as psychotic disorders are incredibly heterogeneous in their symptomology and often present with high levels of comorbidity.^13^, ^14^ For this reason, more often than not, emphasis tends to be focussed on modelling symptoms rather than a disorder per se.^13^ Two ways in which researchers have attempted to create models with face, construct and predictive validity for psychiatric disorders is by reiterating the behavioural and cognitive abnormalities that are seen in the clinical phenotype of the disorder or by mimicking the relevant neural, neurochemical, molecular or anatomical aspects of the disorder in question.^13^ Methods used to create animal models of schizophrenia, especially due to its highly complex and heterogeneous symptomology, can be broadly clustered into 4 different groups: pharmacological‐, genetic‐, developmental‐ and lesion‐induced models.^15^

While there has been no shortage of great appraisals of preclinical models published over the last 2 decades in the form of narrative reviews as a desperate attempt to bring together and try and make sense of this exponentially growing field of research, we believe that a sort of quantification of the animal research field could be profitable in further improving our understanding of the field.

Our aim is to improve our understanding of the role that animal models play in the drug discovery process of psychotic disorders and their validity by providing an unbiased summary of the field. This in turn has the potential to inform the preclinical field of psychosis research on which experiments work best and where improvements can be made for future experiments. A better understanding of the data that exists will inform what we think we know and thus help improve the translation of knowledge between preclinical research and clinical practice.

### 2.2 Research question

#### 2.2.1 Specify the disease/health problem of interest

We are interested in studies looking at non‐affective psychotic disorders, affective psychotic disorders, substance‐induced psychotic disorders and psychotic disorders due to a general medical condition.

#### 2.2.2 Specify the population/species studied

We will not make any exclusions based on species and therefore include studies investigating all animal species except humans.

#### 2.2.3 Specify the intervention/exposure

Our aim is to carry out an exploratory, broad review of the preclinical field of psychosis, and therefore, we are not limiting our review to any particular type of drugs or interventions but will aim to include all interventions described in the literature as a method of model induction for animal models of psychosis. For animal models of schizophrenia, due to the complexity of the clinical profile of the disorder, we expect to come across and therefore will include and differentiate between interventions that can largely be grouped into 4 categories^15^: experiments using genetic, lesion, pharmacological, developmental or a combination of these type of interventions to model aspects recognized to be representative of those in the human disorder.

#### 2.2.4 Specify the control population

For model‐characterizing studies, we recognize a suitable control as an animal that has not been exposed to the method of induction used to create the model itself but has received a sham equivalent where appropriate. Examples of this can be in the form of sham surgery performed on the animal in place of a lesion or the administration of saline or another vehicle used for the dissolution of the active compound given to the model animal.

For treatment intervention studies, we consider a suitable control to be an animal that has had the same exposure to the model of the disorder as those that are given a treatment, but they have not been exposed to the treatment being tested. Here, we will accept both vehicle‐treated and non‐treated animals as an appropriate control.

#### 2.2.5 Specify the outcome measures

We will include studies looking at behavioural, anatomical, electrophysiological and neurochemical outcomes initially to assess the overall quality of studies investigating animal models of psychotic disorders; however, we plan to only extract data for experiments that measure behavioural outcomes at this point in time.

#### 2.2.6 State your research question

We aim to provide an unbiased review of the field, and therefore, our main research question is: what kind of *in vivo* model paradigms are used to model psychotic disorders in animals? Moreover, we are interested in how these experimental models and paradigms are currently being used to measure psychosis‐related outcomes and how they compare in terms of efficiency to modelling the human condition (ie, how good is the face and construct validity of these models?). How well do results in this domain translate to results in human studies, especially in terms of predicting drug efficacy?

#### 2.2.7 Identify literature databases to search

We chose to search the life sciences and biomedical literature database MEDLINE via the search engine PubMed to initially get a shallow, but broad, overview of the field.

#### 2.2.8 Define electronic search strategies

((((((((((((((((((((((((psychot*) OR psychosis) OR psychoses) OR paranoia) OR paraphrenia) OR sensitive beziehungswahn) OR involutional paranoid state) OR folie deux) OR cataton*) OR delusion*) OR hallucinat*) OR schizotyp*) OR psychoactive) OR oneirophrenia) OR psychogen*) OR bouffee delirante) OR hebephrenia) OR schizophren*) OR schizoaffect*) OR manic stupor)) NOT comment*[Publication Type]) NOT case report*[Publication Type]) NOT letter*[Publication Type]) NOT review*[Publication Type]

Please find the full, expanded list of search words in the Supplementary Information.

### 2.3 Study selection

#### 2.3.1 Define screening phases

1. Pre‐screening based on title and abstract.
2. Full‐text screening, categorization and assessment of reporting of experimental risk of bias.
3. Screening at level of data extraction for papers looking at behavioural outcome measures.

The extraction of information regarding the reporting of experimental risk of bias items is simply to obtain an overall picture of the extent that these measures are reported in the literature and will not be used as a tool to exclude papers from further analysis.

#### 2.3.2 Specify (a) the number of reviewers per screening phase and (b) how discrepancies will be resolved

1. Two independent observers.
2. One observer and use of computer‐based data mining as second reviewer for reporting of experimental risk of bias.
3. Primarily one observer but 2 independent observers if possible.

In all phases, discrepancies will be resolved by an independent third reviewer.

### 2.4 Definition of all inclusion and exclusion criteria based on

#### 2.4.1 Type of study (design)

**Inclusion criteria:** primary research articles.

Case reports, human studies, letters or comments, reviews, conference and seminar abstracts without data or instances where data being referred to is not clear from publication and studies where there is no appropriate control group will be excluded.

#### 2.4.2 Type of animals/population

**Inclusion criteria:** Experiments looking at *in vivo*, whole animal models of psychotic disorders that model any symptom recognized to be related to psychotic disorders specified above, including transgenic models of psychotic disorders. Animals of all ages, genders and species will be considered.

Human, ex vivo or in vitro experiments; experiments using animal models of other mental disorders that do not normally feature symptoms of psychosis (ie, affective disorders without psychotic symptoms); and *in vivo* experiments where no disease model is induced before a treatment is tested (ie, pharmacological and toxicity studies examining side effects of anti‐psychotics drugs) will all be excluded.

#### 2.4.3 Type of intervention

**Inclusion criteria:** All types of interventions that aim to model psychotic disorders in animals whether testing a treatment drug or simply characterizing the model. Moreover, we will consider all dosages, durations and frequencies of treatment drug administrations.

Experiments where treatment interventions are given to healthy/wild‐type animals and not animals representing animal models of psychotic disorders (ie, studies that look at pharmacological and toxicity side effects of anti‐psychotics); experiments investigating drug withdrawal in animals; and drug discrimination and other drug addiction testing experiments will be excluded.

#### 2.4.4 Outcome measures

**Inclusion criteria:** In phases 1 and 2, we will include all publications where behavioural, anatomical, electrophysiological and neurochemical outcomes are described. We plan to extract data, foremost, for behavioural outcomes; therefore, publications with all other outcome measures will be retained and analysed for measures reportedly taken to reduce the risk of experimental bias but will not be taken forward for meta‐analysis at this stage (phase 3).

Any other outcome measures, such as metabolic outcomes or genetic analyses, will not be included.

#### 2.4.5 Language restrictions

**Inclusion criteria:** all languages.

**Exclusion criteria:** none.

#### 2.4.6 Publication date restrictions

**Inclusion criteria:** all dates.

**Exclusion criteria:** none.

#### 2.4.7 Sort and prioritize your exclusion criteria per selection phase

We plan to exclude papers based on the following criteria at the following 3 stages of the review, split into a list of exclusion criteria listed in order of importance:

**Selection phase 1: Screening on basis of title and abstract**

1. Not a primary research article (i.e. review, comment, editorial, letter to the editor).
2. Study involving human participants and no animal data in publication.
3. In vitro models.
4. No psychosis or aspect of schizophrenia induced.
5. No appropriate outcome measure (behavioural, anatomical, electrophysiological or neurochemical).

All papers not excluded by the inclusion criteria above then go on to phase 2 of the project.

**Selection phase 2: Initial full‐text screening**

1. No induction of animal model of psychotic disorders.
2. If only behavioural outcomes reported; no outcomes measured thought to be relevant to psychosis.

Papers not excluded by the inclusion criteria above go on to phase 3 of the project. At this stage, we will exclude papers not looking at behavioural measures from further stages of the review; however, we will not be excluding papers based on their score in our assessment of the reporting of measures taken to reduce the risk of bias.

**Selection phase 3: Final inclusion for data extraction and meta‐analysis**

1. No behavioural outcome measure reported.
2. No appropriate control.
3. No n numbers or SD/SEM provided.

Papers not meeting the inclusion criteria at this phase of the project will be excluded from the meta‐analysis but will remain part of the overall review of the field that describes the type of animal models used, the types of outcomes assessed and the overall prevalence of reporting of measures taken to reduce the risk of bias.

### 2.5 Study characteristics to be extracted (for assessment of external validity, reporting quality)

#### 2.5.1 Study ID

The following data specific to each study will be extracted: name of first and corresponding authors, year of publication, title and journal name.

#### 2.5.2 Study design characteristics

Number of animals in control and experimental groups will be extracted. If *n* numbers are given as a range, the most conservative estimate will be extracted.

#### 2.5.3 Animal model characteristics

If reported, we will collect data on the following details about animals used in study: species, strain, gender, age at time of testing and weight. Furthermore, information on which specific human disorder the animal is considered to be modelling and details about the method of disorder induction (including if transgenic and if a combination model) will be extracted. Time elapsed between model induction and outcome measurement is also of interest and will be recorded.

#### 2.5.4 Intervention characteristics

We will extract information about the exact treatment tested, dosage and mode of delivery of this treatment, as well as time when treatment is administered, frequency of treatment administration and length of treatment course. Time elapsed between treatment administration and outcome measurement will also be noted.

#### 2.5.5 Outcome measures

Of interest to us are the types of outcome measures that are measured in a study, for behavioural outcomes more specifically: the exact name of each outcome that is measured and what animal behaviour outcome measure is intended to quantify. Furthermore, for each outcome, we will also extract the mean, SD or SEM and *n* for both the control and experimental groups.

#### 2.5.6 Other

Where reported, we will also extract data on the number of excluded animals and reason given, if any, for exclusion.

### 2.6 Assessment of the reporting of measures taken to reduce the risk of bias (internal validity)

(a) The number of reviewers assessing the reporting of risk of bias in each study and (b) how discrepancies will be resolved.

- Risk of bias will be evaluated by scoring the reporting of measures taken to reduce the risk of experimental bias within studies, primarily by a single investigator and secondarily using computer‐based data mining tools.
- Discrepancies will be resolved by a second independent reviewer.

#### 2.6.1 Criteria to assess the internal validity of included studies

Reporting of measures taken to reduce the risk of bias will be assessed using the previously used CAMARADES study quality checklist,^16^ adapted to include the following items:

1. Reporting of randomization.
2. Evidence of blinded conduct of experiment.
3. Evidence of blinded assessment of outcome.
4. Statement of inclusion and exclusion criteria.
5. Reporting of sample size calculation.
6. Statement of possible conflict of interest.
7. Statement of compliance with animal welfare regulations.
8. Availability of a study protocol.

### 2.7 Collection of outcome data

#### 2.7.1 For each outcome measure, define the type of data to be extracted

We will extract all relevant comparisons of behavioural outcomes within a study, including where animals of different ages, genders or species are used. We expect this to be continuous data. We will extract data separately for comparisons where any of these variables differ in the group of animals being analysed from another comparison. We will also separately extract data for outcomes measured as a result of different treatment regimens. For each outcome measure, mean, SD or SEM and *n* numbers will be extracted for both experimental and control groups.

#### 2.7.2 Methods for data extraction/retrieval

We will preferably and primarily extract numerical data from the text of each publication (including if presented in a tabular format). In studies where data are only presented graphically, the Adobe Reader Measuring Tool in Adobe Acrobat XI will be used to extract numerical data. If any of the data we are interested in are unclear from the publication or missing, we plan to contact authors of the publication by email to obtain the correct data. In the absence of a response from authors, data will be excluded from analysis.

#### 2.7.3 Specify (a) the number of reviewers extracting data and (b) how discrepancies will be resolved

- Number of reviewers extracting data will primarily be one, but 2 if resources allow.
- Any discrepancies would be resolved by a third independent reviewer.

#### 2.7.4 Specify (per outcome measure) how you are planning to combine/compare the data

We plan on aggregating the data using a meta‐analysis with subgroup analyses separately for model characterizing and treatment‐exploring studies. When it comes to analysing the data, where data are reported from independent groups of animals, we will treat data reported as independent comparisons and will include them separately in our meta‐analysis. Where multiple behavioural outcomes are reported from the same cohort of animals, we will nest data using the fixed‐effects model. This will be performed separately for comparisons looking at the performance of a model and comparing it to a naïve animal and those where a treatment is being tested. Where a control group serves more than one experimental group within a study, we will correct the number of animals that are calculated in our meta‐analysis by dividing the number of animals that is reported in the control group by the number of groups it serves within the study. As we are interested in behavioural outcomes and how these change over a period of time, we will calculate area under the curve for different comparisons and will use data at all reported time points to calculate an overall estimate for that comparison.^17^

#### 2.7.5 Specify (per outcome measure) how it will be decided whether a meta‐analysis will be performed

We consider the performance of a meta‐analysis appropriate in the case where we have more than 10 outcomes for an outcome measure.

### 2.8 If a meta‐analysis seems feasible/sensible, specify (for each outcome measure)

We will analyse studies characterizing the model and those looking at the efficacy of a therapeutic intervention separately. The method used for analysis will be identical for both, but we will look at different study characteristics when assessing for potential sources of heterogeneity.

#### 2.8.1 The effect measure to be used

Where the performance of a normal, unlesioned, wild‐type animal is known or can be inferred in at least 80% of experiments, we will use normalized mean difference meta‐analysis as the primary outcome, with standardized mean difference as a sensitivity analysis. If the performance of a normal, unlesioned, wild‐type animal is unknown or can be inferred in less than 80% of experiments, we will use standardized a mean difference meta‐analysis as the primary outcome, with normalized mean difference as a sensitivity analysis. We will combine multiple outcomes from the same experimental cohorts using fixed‐effects meta‐analysis.^17^

#### 2.8.2 The statistical model of analysis

We will use the random‐effects model to pool and analyse effect sizes from different comparisons as we expect there to be a considerable amount of heterogeneity between studies due to the large diversity in study design. This will thus take into account both within‐study (sampling error) as well as between‐study variance.^17^ While the meta‐analysis will provide a global estimate of efficacy of anti‐psychotic agents, this is of very limited utility, and a much more important finding is evidence for any heterogeneity to more closely identify which aspects of anti‐psychotic treatments and experimental designs are associated with different levels of efficacy.

#### 2.8.3 The statistical methods to assess heterogeneity

**Which study characteristics will be examined as potential source of heterogeneity?**

We will assess differences between the reporting of risk of bias quality assessment subgroups, including each individual quality item and number of study quality checklist items scored by publication.
We also plan to assess differences between study characteristics assessment subgroups, which will be different for model characterizing and treatment exploring experiments (Table 2).

**Table 2:** Study characteristics assessment subgroups that will be examined as potential sources of heterogeneity

**Model characterizing studies**	**Treatment exploring studies**
Species of animals used	Species of animals used
Gender of animals used	Gender of animals used
Specific intervention used to induce model	Specific intervention used to induce model
For schizophrenia models: Method of model induction (ie, developmental, genetic, pharmacological, lesion or combination)	For schizophrenia models: Method of model induction (ie, developmental, genetic, pharmacological, lesion or combination)
Extent of lesion/dose of drug used to induce model	Extent of lesion/dose of drug used to induce model
Outcome being measured	Outcome being measured
Exact methods used to assess outcome measure	Exact methods used to assess outcome measure
Time of outcome measurement (in relation to model induction)	Time of outcome measurement (in relation to model induction and/or treatment administration)
	Treatment given
	Dose of treatment given
	Time of administration

#### 2.8.4 Any sensitivity analyses you propose to perform

Where we use normalized mean difference meta‐analysis as the primary outcome, we will use standardized mean difference as a sensitivity analysis. Where we use standardized mean difference meta‐analysis as the primary outcome, we will use normalized mean difference as a sensitivity analysis.

#### 2.8.5 Other details of the meta‐analysis

For partitioning of heterogeneity, we will use the Holm‐Bonferroni method to adjust critical values of *P* separately for quality items and study design items in subgroup analyses.

#### 2.8.6 The method for assessment of publication bias

Risk of publication bias will be evaluated using funnel plot assessment and Egger's regression.^18^, ^19^ Trim and fill analysis^20^ using STATA will be used to identify possible missing studies in the literature. These evaluations will be conducted independently for each outcome measure using non‐nested data.

## Supporting information


**Appendix S1**. Full list of search words as expanded by PubMed.
