# Supplementary material for: Improving our understanding of the in vivo modelling of psychotic disorders: A protocol for a systematic review and meta‐analysis
Source: Evid Based Preclin Med. 2017 Mar 17;3(2):e00022. doi: 10.1002/ebm2.22 (PMC5367269; doi:10.1002/ebm2.22)
Supplement: Supplementary file 1 — Appendix S1. Full list of search words as expanded by PubMed. [file EBM2-3-10-s001.docx]

**Full list of search words as expanded by PubMed:**

(((((((((((((((((((((((psychot[All Fields] OR psychot'erapie[All Fields] OR psychotactile[All Fields] OR psychotainment[All Fields] OR psychotaxonomy[All Fields] OR psychote'erapeutique[All Fields] OR psychotechnic[All Fields] OR psychotechnical[All Fields] OR psychotechnicians[All Fields] OR psychotechnicien[All Fields] OR psychotechniciens[All Fields] OR psychotechnics[All Fields] OR psychotechnie[All Fields] OR psychotechnik[All Fields] OR psychotechniken[All Fields] OR psychotechnique[All Fields] OR psychotechniques[All Fields] OR psychotechnisch[All Fields] OR psychotechnische[All Fields] OR psychotechnischen[All Fields] OR psychotechnischer[All Fields] OR psychotechnological[All Fields] OR psychotechnologies[All Fields] OR psychotechnologies'[All Fields] OR psychotechnologists[All Fields] OR psychotechnology[All Fields] OR psychotehnic[All Fields] OR psychotehrapies[All Fields] OR psychotemporal[All Fields] OR psychoten[All Fields] OR psychoterapei[All Fields] OR psychoterapeuta[All Fields] OR psychoterapeutic[All Fields] OR psychoterapeutick[All Fields] OR psychoterapeutick'e[All Fields] OR psychoterapeutick'y[All Fields] OR psychoterapeuticka[All Fields] OR psychoterapeuticke[All Fields] OR psychoterapeutickeho[All Fields] OR psychoterapeutickej[All Fields] OR psychoterapeutickem[All Fields] OR psychoterapeutickom[All Fields] OR psychoterapeuticky[All Fields] OR psychoterapeutickych[All Fields] OR psychoterapeutische[All Fields] OR psychoterapeutom[All Fields] OR psychoterapeutova[All Fields] OR psychoterapeuty[All Fields] OR psychoterapeutyczne[All Fields] OR psychoterapeutycznego[All Fields] OR psychoterapeutycznej[All Fields] OR psychoterapeutycznych[All Fields] OR psychoteraph[All Fields] OR psychoteraphy[All Fields] OR psychoteraphy'[All Fields] OR psychoterapia[All Fields] OR psychoterapic[All Fields] OR psychoterapie[All Fields] OR psychoterapii[All Fields] OR psychoterapique[All Fields] OR psychoterapist[All Fields] OR psychoterapists[All Fields] OR psychoterapli[All Fields] OR psychoterappi[All Fields] OR psychoterapy[All Fields] OR psychoteratogenesis[All Fields] OR psychoteratogenic[All Fields] OR psychoteratogenicity[All Fields] OR psychoteratology[All Fields] OR psychoterminal[All Fields] OR psychoterminalny[All Fields] OR psychoterror[All Fields] OR psychotest[All Fields] OR psychotestings[All Fields] OR psychotestu[All Fields] OR psychotetramine[All Fields] OR psychoth[All Fields] OR psychoth'erapeutique[All Fields] OR psychoth'erapie[All Fields] OR psychoth'erapies[All Fields] OR psychoth'erapique[All Fields] OR psychoth'erapiques[All Fields] OR psychoth6rapeute[All Fields] OR psychotheapeutic[All Fields] OR psychotheapy[All Fields] OR psychotheeapy[All Fields] OR psychothematics[All Fields] OR psychothematik[All Fields] OR psychotheological[All Fields] OR psychotheoretical[All Fields] OR psychother[All Fields] OR psychothera[All Fields] OR psychotheraeuten[All Fields] OR psychotheraia[All Fields] OR psychotheraoeutische[All Fields] OR psychotherap[All Fields] OR psychotherapei[All Fields] OR psychotherapeia[All Fields] OR psychotherapeieresultaten[All Fields] OR psychotherapeifallen[All Fields] OR psychotherapetic[All Fields] OR psychotherapeut[All Fields] OR psychotherapeute[All Fields] OR psychotherapeuten[All Fields] OR psychotherapeutenausbildung[All Fields] OR psychotherapeutengesetz[All Fields] OR psychotherapeutengesetzes[All Fields] OR psychotherapeutenverhaltens[All Fields] OR psychotherapeutes[All Fields] OR psychotherapeutic[All Fields] OR psychotherapeutic'[All Fields] OR psychotherapeutical[All Fields] OR psychotherapeutically[All Fields] OR psychotherapeuticapproach[All Fields] OR psychotherapeutiche[All Fields] OR psychotherapeutickeho[All Fields] OR psychotherapeutics[All Fields] OR psychotherapeuties[All Fields] OR psychotherapeutik[All Fields] OR psychotherapeutikum[All Fields] OR psychotherapeutin[All Fields] OR psychotherapeutinnen[All Fields] OR psychotherapeutinuniversitatsklinik[All Fields] OR psychotherapeutique[All Fields] OR psychotherapeutiques[All Fields] OR psychotherapeutis[All Fields] OR psychotherapeutisch[All Fields] OR psychotherapeutische[All Fields] OR psychotherapeutischem[All Fields] OR psychotherapeutischen[All Fields] OR psychotherapeutischer[All Fields] OR psychotherapeutisches[All Fields] OR psychotherapeutist[All Fields] OR psychotherapeutists[All Fields] OR psychotherapeutization[All Fields] OR psychotherapeuts[All Fields] OR psychotherapeuty[All Fields] OR psychotheraphie[All Fields] OR psychotheraphobia[All Fields] OR psychotheraphy[All Fields] OR psychotherapi[All Fields] OR psychotherapi'aj'anak[All Fields] OR psychotherapia[All Fields] OR psychotherapiaban[All Fields] OR psychotherapias[All Fields] OR psychotherapic[All Fields] OR psychotherapicabteilung[All Fields] OR psychotherapie[All Fields] OR psychotherapieabteilung[All Fields] OR psychotherapieakzeptanz[All Fields] OR psychotherapiealternative[All Fields] OR psychotherapieangebots[All Fields] OR psychotherapieaschaffenburg[All Fields] OR psychotherapieassistenten[All Fields] OR psychotherapieauffassung[All Fields] OR psychotherapieausbildung[All Fields] OR psychotherapiebedurfnis[All Fields] OR psychotherapiebedurftiger[All Fields] OR psychotherapiebedurftigkeit[All Fields] OR psychotherapiebegleitende[All Fields] OR psychotherapiedokumentation[All Fields] OR psychotherapieeffekte[All Fields] OR psychotherapieempfehlungen[All Fields] OR psychotherapieerfolg[All Fields] OR psychotherapieerfolges[All Fields] OR psychotherapieergebnissen[All Fields] OR psychotherapieform[All Fields] OR psychotherapieformen[All Fields] OR psychotherapieforscher[All Fields] OR psychotherapieforschung[All Fields] OR psychotherapiegruppen[All Fields] OR psychotherapieindikation[All Fields] OR psychotherapieinduzierter[All Fields] OR psychotherapieinstitution[All Fields] OR psychotherapieklinik[All Fields] OR psychotherapiekongress[All Fields] OR psychotherapiekontrolle[All Fields] OR psychotherapiekonzept[All Fields] OR psychotherapiemethoden[All Fields] OR psychotherapiemotivation[All Fields] OR psychotherapien[All Fields] OR psychotherapiepatienten[All Fields] OR psychotherapiepatientinnen[All Fields] OR psychotherapieplanung[All Fields] OR psychotherapieplatz[All Fields] OR psychotherapiepraxis[All Fields] OR psychotherapieprinzip[All Fields] OR psychotherapieprogramme[All Fields] OR psychotherapieprozessforschung[All Fields] OR psychotherapierelevanten[All Fields] OR psychotherapierichtlinien[All Fields] OR psychotherapies[All Fields] OR psychotherapies'[All Fields] OR psychotherapieschulen[All Fields] OR psychotherapiestation[All Fields] OR psychotherapiestationen[All Fields] OR psychotherapiestudie[All Fields] OR psychotherapiestudien[All Fields] OR psychotherapieverfahren[All Fields] OR psychotherapieverlauf[All Fields] OR psychotherapieverlaufe[All Fields] OR psychotherapieverlaufsforschung[All Fields] OR psychotherapieversuch[All Fields] OR psychotherapiewoche[All Fields] OR psychotherapii[All Fields] OR psychotherapiqe[All Fields] OR psychotherapique[All Fields] OR psychotherapiques[All Fields] OR psychotherapist[All Fields] OR psychotherapist'[All Fields] OR psychotherapist's[All Fields] OR psychotherapiste[All Fields] OR psychotherapists[All Fields] OR psychotherapists'[All Fields] OR psychotherapits[All Fields] OR psychotherapty[All Fields] OR psychotherapuetic[All Fields] OR psychotheraputic[All Fields] OR psychotherapy[All Fields] OR psychotherapy'[All Fields] OR psychotherapy's[All Fields] OR psychotherapy1[All Fields] OR psychotherapygenetics[All Fields] OR psychotherapyuniversity[All Fields] OR psychotherapyw2[All Fields] OR psychotheratpie[All Fields] OR psychotheray[All Fields] OR psychotherayp[All Fields] OR psychothereutic[All Fields] OR psychotheroapy[All Fields] OR psychotherories[All Fields] OR psychotherpay[All Fields] OR psychotherpeutic[All Fields] OR psychotherpie[All Fields] OR psychotherpiemotivation[All Fields] OR psychotherpists[All Fields] OR psychotherpy[All Fields] OR psychotherrapeutic[All Fields] OR psychotherspy[All Fields] OR psychothrapeutic[All Fields] OR psychothraphy[All Fields] OR psychothrapie[All Fields] OR psychothropic[All Fields] OR psychothymic[All Fields] OR psychotic[All Fields] OR psychotic'[All Fields] OR psychotic's[All Fields] OR psychotica[All Fields] OR psychotical[All Fields] OR psychotically[All Fields] OR psychoticbody[All Fields] OR psychotici[All Fields] OR psychoticism[All Fields] OR psychoticism'[All Fields] OR psychoticismu[All Fields] OR psychoticity[All Fields] OR psychoticity'[All Fields] OR psychoticizm[All Fields] OR psychotick'y[All Fields] OR psychotick'ym[All Fields] OR psychoticka[All Fields] OR psychoticke[All Fields] OR psychotickeho[All Fields] OR psychoticky[All Fields] OR psychotickych[All Fields] OR psychoticlike[All Fields] OR psychoticng[All Fields] OR psychotics[All Fields] OR psychotics'[All Fields] OR psychoticum[All Fields] OR psychotiform[All Fields] OR psychotiform'[All Fields] OR psychotigues[All Fields] OR psychotiker[All Fields] OR psychotikern[All Fields] OR psychotikmi[All Fields] OR psychotiku[All Fields] OR psychotikum[All Fields] OR psychotikus[All Fields] OR psychotikusokon[All Fields] OR psychotiky[All Fields] OR psychotique[All Fields] OR psychotiques[All Fields] OR psychotisation[All Fields] OR psychotisch[All Fields] OR psychotische[All Fields] OR psychotische'[All Fields] OR psychotischem[All Fields] OR psychotischen[All Fields] OR psychotischer[All Fields] OR psychotisches[All Fields] OR psychotisicm[All Fields] OR psychotism[All Fields] OR psychotizing[All Fields] OR psychotizismus[All Fields] OR psychoto[All Fields] OR psychotocism[All Fields] OR psychotoform[All Fields] OR psychotogen[All Fields] OR psychotogenesis[All Fields] OR psychotogenic[All Fields] OR psychotogenicity[All Fields] OR psychotogens[All Fields] OR psychotogique[All Fields] OR psychotogiques[All Fields] OR psychotoid[All Fields] OR psychotoid'[All Fields] OR psychotoksykologii[All Fields] OR psychotolysis[All Fields] OR psychotolytic[All Fields] OR psychotomimesis[All Fields] OR psychotomimetic[All Fields] OR psychotomimetic'[All Fields] OR psychotomimetically[All Fields] OR psychotomimetics[All Fields] OR psychotomimeticumok[All Fields] OR psychotomimetika[All Fields] OR psychotomimetique[All Fields] OR psychotomimetiques[All Fields] OR psychotomimetische[All Fields] OR psychotomimetric[All Fields] OR psychotomimmetic[All Fields] OR psychotomorphic[All Fields] OR psychotomymetic[All Fields] OR psychoton[All Fields] OR psychotone[All Fields] OR psychotonic[All Fields] OR psychotonica[All Fields] OR psychotonics[All Fields] OR psychotonicumok[All Fields] OR psychotonika[All Fields] OR psychotonikum[All Fields] OR psychotonikums[All Fields] OR psychotonin[All Fields] OR psychotonique[All Fields] OR psychotoniques[All Fields] OR psychotonisierender[All Fields] OR psychotonovy[All Fields] OR psychotonu[All Fields] OR psychotop[All Fields] OR psychotopes[All Fields] OR psychotopic[All Fields] OR psychotopics[All Fields] OR psychotorpnijch[All Fields] OR psychototmimetic[All Fields] OR psychotoxic[All Fields] OR psychotoxical[All Fields] OR psychotoxicity[All Fields] OR psychotoxicologic[All Fields] OR psychotoxicological[All Fields] OR psychotoxicology[All Fields] OR psychotoxikologie[All Fields] OR psychotoxikologische[All Fields] OR psychotoxin[All Fields] OR psychotoxins[All Fields] OR psychotoxischen[All Fields] OR psychotraining[All Fields] OR psychotramide[All Fields] OR psychotrauma[All Fields] OR psychotrauma's[All Fields] OR psychotraumagroep[All Fields] OR psychotraumas[All Fields] OR psychotraumas'[All Fields] OR psychotraumata[All Fields] OR psychotraumatic[All Fields] OR psychotraumatique[All Fields] OR psychotraumatiques[All Fields] OR psychotraumatischen[All Fields] OR psychotraumatised[All Fields] OR psychotraumatism[All Fields] OR psychotraumatisme[All Fields] OR psychotraumatismes[All Fields] OR psychotraumatitisierter[All Fields] OR psychotraumatizace[All Fields] OR psychotraumatization[All Fields] OR psychotraumatized[All Fields] OR psychotraumatizing[All Fields] OR psychotraumatol[All Fields] OR psychotraumatologi[All Fields] OR psychotraumatological[All Fields] OR psychotraumatologie[All Fields] OR psychotraumatologische[All Fields] OR psychotraumatologischen[All Fields] OR psychotraumatologischer[All Fields] OR psychotraumatology[All Fields] OR psychotria[All Fields] OR psychotriae[All Fields] OR psychotrianoside[All Fields] OR psychotrianosides[All Fields] OR psychotrichological[All Fields] OR psychotrichology[All Fields] OR psychotridine[All Fields] OR psychotrieae[All Fields] OR psychotriifolia[All Fields] OR psychotrimine[All Fields] OR psychotrine[All Fields] OR psychotripine[All Fields] OR psychotroic[All Fields] OR psychotronic[All Fields] OR psychotronice[All Fields] OR psychotronics[All Fields] OR psychotronika[All Fields] OR psychotrop[All Fields] OR psychotropa[All Fields] OR psychotrope[All Fields] OR psychotropen[All Fields] OR psychotroper[All Fields] OR psychotropes[All Fields] OR psychotroph[All Fields] OR psychotrophes[All Fields] OR psychotrophic[All Fields] OR psychotrophics[All Fields] OR psychotrophie[All Fields] OR psychotrophs[All Fields] OR psychotropia[All Fields] OR psychotropic[All Fields] OR psychotropic'[All Fields] OR psychotropic's[All Fields] OR psychotropica[All Fields] OR psychotropical[All Fields] OR psychotropically[All Fields] OR psychotropicity[All Fields] OR psychotropicmedication[All Fields] OR psychotropics[All Fields] OR psychotropics'[All Fields] OR psychotropid[All Fields] OR psychotropism[All Fields] OR psychotropn'ich[All Fields] OR psychotropne[All Fields] OR psychotropneho[All Fields] OR psychotropni[All Fields] OR psychotropnich[All Fields] OR psychotropnimi[All Fields] OR psychotropowe[All Fields] OR psychotropowego[All Fields] OR psychotropowych[All Fields] OR psychotropowym[All Fields] OR psychotropowymi[All Fields] OR psychotrops[All Fields] OR psychotropy[All Fields] OR psychotroropes[All Fields] OR psychotrpic[All Fields] OR psychotuberculosis[All Fields] OR psychotychznych[All Fields] OR psychotyczna[All Fields] OR psychotyczne[All Fields] OR psychotycznego[All Fields] OR psychotycznej[All Fields] OR psychotyczny[All Fields] OR psychotycznych[All Fields] OR psychotycznym[All Fields] OR psychotycznymi[All Fields] OR psychotyczych[All Fields] OR psychotype[All Fields] OR psychotypes[All Fields] OR psychotypical[All Fields] OR psychotypological[All Fields] OR psychotypology[All Fields] OR psychotyzcznymi[All Fields] OR psychotyzm[All Fields]) OR ("psychotic disorders"[MeSH Terms] OR ("psychotic"[All Fields] AND "disorders"[All Fields]) OR "psychotic disorders"[All Fields] OR "psychosis"[All Fields])) OR ("psychotic disorders"[MeSH Terms] OR ("psychotic"[All Fields] AND "disorders"[All Fields]) OR "psychotic disorders"[All Fields] OR "psychoses"[All Fields])) OR ("paranoid disorders"[MeSH Terms] OR ("paranoid"[All Fields] AND "disorders"[All Fields]) OR "paranoid disorders"[All Fields] OR "paranoia"[All Fields])) OR paraphrenia[All Fields]) OR (sensitive[All Fields] AND beziehungswahn[All Fields])) OR (involutional[All Fields] AND paranoid[All Fields] AND state[All Fields])) OR ("shared paranoid disorder"[MeSH Terms] OR ("shared"[All Fields] AND "paranoid"[All Fields] AND "disorder"[All Fields]) OR "shared paranoid disorder"[All Fields] OR ("folie"[All Fields] AND "deux"[All Fields]) OR "folie deux"[All Fields])) OR (catatonia[All Fields] OR catatonia'[All Fields] OR catatonialike[All Fields] OR catatonias[All Fields] OR catatonic[All Fields] OR catatonica[All Fields] OR catatonical[All Fields] OR catatoniche[All Fields] OR catatonici[All Fields] OR catatoniclike[All Fields] OR catatonico[All Fields] OR catatonicocatalettiche[All Fields] OR catatonicos[All Fields] OR catatonics[All Fields] OR catatonie[All Fields] OR catatonies[All Fields] OR catatoniform[All Fields] OR catatoniformpsychomotor[All Fields] OR catatonig'ene[All Fields] OR catatonigene[All Fields] OR catatonigenes[All Fields] OR catatonigenic[All Fields] OR catatonin[All Fields] OR catatonine[All Fields] OR catatonique[All Fields] OR catatoniques[All Fields] OR catatonis[All Fields] OR catatonizante[All Fields] OR catatonizzati[All Fields] OR catatono[All Fields] OR catatonogenic[All Fields] OR catatonoid[All Fields] OR catatony[All Fields])) OR (delusion[All Fields] OR delusion'[All Fields] OR delusion's[All Fields] OR delusional[All Fields] OR delusional'[All Fields] OR delusionalilty[All Fields] OR delusionalism[All Fields] OR delusionality[All Fields] OR delusionally[All Fields] OR delusionals[All Fields] OR delusionary[All Fields] OR delusione[All Fields] OR delusiones[All Fields] OR delusioni[All Fields] OR delusionlike[All Fields] OR delusionnal[All Fields] OR delusions[All Fields] OR delusions'[All Fields])) OR (hallucinate[All Fields] OR hallucinated[All Fields] OR hallucinates[All Fields] OR hallucinatic[All Fields] OR hallucinatie[All Fields] OR hallucinaties[All Fields] OR hallucinatiion[All Fields] OR hallucinatiions[All Fields] OR hallucinating[All Fields] OR hallucination[All Fields] OR hallucination'[All Fields] OR hallucinational[All Fields] OR hallucinationen[All Fields] OR hallucinationer[All Fields] OR hallucinationes[All Fields] OR hallucinations[All Fields] OR hallucinations'[All Fields] OR hallucinations'than[All Fields] OR hallucinatire[All Fields] OR hallucinative[All Fields] OR hallucinatoire[All Fields] OR hallucinatoire'[All Fields] OR hallucinatoires[All Fields] OR hallucinatoiresaigues[All Fields] OR hallucinator[All Fields] OR hallucinatoria[All Fields] OR hallucinatoric[All Fields] OR hallucinatornih[All Fields] OR hallucinators[All Fields] OR hallucinators'[All Fields] OR hallucinatory[All Fields] OR hallucinatory'[All Fields] OR hallucinatroy[All Fields])) OR (schizotypai[All Fields] OR schizotypal[All Fields] OR schizotypal'[All Fields] OR schizotypal''[All Fields] OR schizotypality[All Fields] OR schizotypals[All Fields] OR schizotype[All Fields] OR schizotype's[All Fields] OR schizotypes[All Fields] OR schizotypes'[All Fields] OR schizotypia[All Fields] OR schizotypic[All Fields] OR schizotypical[All Fields] OR schizotypics[All Fields] OR schizotypie[All Fields] OR schizotypies[All Fields] OR schizotypique[All Fields] OR schizotypische[All Fields] OR schizotypischen[All Fields] OR schizotypital[All Fields] OR schizotypy[All Fields] OR schizotypy'[All Fields] OR schizotypyal[All Fields] OR schizotypys[All Fields])) OR psychoactive[All Fields]) OR oneirophrenia[All Fields]) OR (psychogen[All Fields] OR psychogender[All Fields] OR psychogene[All Fields] OR psychogeneic[All Fields] OR psychogenem[All Fields] OR psychogenen[All Fields] OR psychogenenic[All Fields] OR psychogeneous[All Fields] OR psychogener[All Fields] OR psychogenes[All Fields] OR psychogenese[All Fields] OR psychogenesis[All Fields] OR psychogenetic[All Fields] OR psychogenetical[All Fields] OR psychogenetically[All Fields] OR psychogenetics[All Fields] OR psychogenetique[All Fields] OR psychogenetiques[All Fields] OR psychogenetische[All Fields] OR psychogenetycznym[All Fields] OR psychogenia[All Fields] OR psychogenias[All Fields] OR psychogeniatrie[All Fields] OR psychogeniatrie'[All Fields] OR psychogenic[All Fields] OR psychogenic'[All Fields] OR psychogenica[All Fields] OR psychogenical[All Fields] OR psychogenically[All Fields] OR psychogenicity[All Fields] OR psychogenicor[All Fields] OR psychogenics[All Fields] OR psychogenie[All Fields] OR psychogeniecharakterliche[All Fields] OR psychogenien[All Fields] OR psychogenies[All Fields] OR psychogenique[All Fields] OR psychogeniques[All Fields] OR psychogenitat[All Fields] OR psychogenitically[All Fields] OR psychogenna[All Fields] OR psychogenne[All Fields] OR psychogennego[All Fields] OR psychogenneho[All Fields] OR psychogennej[All Fields] OR psychogenni[All Fields] OR psychogennich[All Fields] OR psychogennie[All Fields] OR psychogenniho[All Fields] OR psychogennim[All Fields] OR psychogennimi[All Fields] OR psychogenny[All Fields] OR psychogennych[All Fields] OR psychogennym[All Fields] OR psychogennymi[All Fields] OR psychogenomic[All Fields] OR psychogenomics[All Fields] OR psychogenous[All Fields] OR psychogenously[All Fields] OR psychogenuously[All Fields] OR psychogeny[All Fields])) OR (bouffee[All Fields] AND delirante[All Fields])) OR ("schizophrenia, disorganized"[MeSH Terms] OR ("schizophrenia"[All Fields] AND "disorganized"[All Fields]) OR "disorganized schizophrenia"[All Fields] OR "hebephrenia"[All Fields])) OR (schizophren[All Fields] OR schizophrena[All Fields] OR schizophrenc[All Fields] OR schizophrenci[All Fields] OR schizophrencis[All Fields] OR schizophrencs[All Fields] OR schizophrene[All Fields] OR schizophrene's[All Fields] OR schizophrenek[All Fields] OR schizophrenem[All Fields] OR schizophrenen[All Fields] OR schizophrenengruppe[All Fields] OR schizophrenenproblem[All Fields] OR schizophrener[All Fields] OR schizophrenes[All Fields] OR schizophrenese[All Fields] OR schizophreni[All Fields] OR schizophreni'as[All Fields] OR schizophrenia[All Fields] OR schizophrenia'[All Fields] OR schizophrenia's[All Fields] OR schizophrenia1[All Fields] OR schizophreniaban[All Fields] OR schizophreniac[All Fields] OR schizophreniaclinicaltrials[All Fields] OR schizophreniacs[All Fields] OR schizophreniaes[All Fields] OR schizophreniaforum[All Fields] OR schizophreniagene[All Fields] OR schizophreniagroup[All Fields] OR schizophreniai[All Fields] OR schizophreniak[All Fields] OR schizophrenial[All Fields] OR schizophrenialike[All Fields] OR schizophreniaor[All Fields] OR schizophreniara[All Fields] OR schizophreniaresearch[All Fields] OR schizophreniaresearchforum[All Fields] OR schizophreniarol[All Fields] OR schizophrenias[All Fields] OR schizophrenias'[All Fields] OR schizophreniaspectrum[All Fields] OR schizophreniay[All Fields] OR schizophrenic[All Fields] OR schizophrenic'[All Fields] OR schizophrenic's[All Fields] OR schizophrenical[All Fields] OR schizophrenically[All Fields] OR schizophrenices[All Fields] OR schizophrenicity[All Fields] OR schizophreniclike[All Fields] OR schizophrenicpatients[All Fields] OR schizophrenics[All Fields] OR schizophrenics'[All Fields] OR schizophrenicss[All Fields] OR schizophrenie[All Fields] OR schizophrenie'[All Fields] OR schizophrenieaehnlichen[All Fields] OR schizophrenieahnliche[All Fields] OR schizophrenieahnlichen[All Fields] OR schizophrenieahnlicher[All Fields] OR schizophrenieartige[All Fields] OR schizophrenieartigen[All Fields] OR schizophrenieartiger[All Fields] OR schizophreniebeggriffs[All Fields] OR schizophreniebegriff[All Fields] OR schizophreniebegriffes[All Fields] OR schizophreniebegriffs[All Fields] OR schizophreniebehandlung[All Fields] OR schizophreniebehandlungen[All Fields] OR schizophreniediagnose[All Fields] OR schizophrenieerkrankten[All Fields] OR schizophrenieerkrankter[All Fields] OR schizophreniefalle[All Fields] OR schizophreniefallen[All Fields] OR schizophrenieform[All Fields] OR schizophrenieforme[All Fields] OR schizophrenieformen[All Fields] OR schizophrenieformer[All Fields] OR schizophrenieforschung[All Fields] OR schizophreniefrage[All Fields] OR schizophreniegenese[All Fields] OR schizophreniekonzepte[All Fields] OR schizophreniekonzepten[All Fields] OR schizophreniekranke[All Fields] OR schizophreniekranken[All Fields] OR schizophreniekranker[All Fields] OR schizophreniekreises[All Fields] OR schizophrenielehre[All Fields] OR schizophrenien[All Fields] OR schizophrenienahe[All Fields] OR schizophreniepatienten[All Fields] OR schizophreniepatientinnen[All Fields] OR schizophrenieproblem[All Fields] OR schizophrenieproblems[All Fields] OR schizophrenierisiko[All Fields] OR schizophrenieritoriality[All Fields] OR schizophrenies[All Fields] OR schizophreniespektrums[All Fields] OR schizophreniespezifitat[All Fields] OR schizophreniestudie[All Fields] OR schizophreniesymptomen[All Fields] OR schizophrenietheorie[All Fields] OR schizophrenietherapie[All Fields] OR schizophrenietypologie[All Fields] OR schizophrenieverlauf[All Fields] OR schizophrenieverlaufe[All Fields] OR schizophrenieverlaufs[All Fields] OR schizophrenieverstandnis[All Fields] OR schizophrenifallen[All Fields] OR schizophreniforems[All Fields] OR schizophreniform[All Fields] OR schizophreniform'[All Fields] OR schizophreniforme[All Fields] OR schizophreniformen[All Fields] OR schizophreniformes[All Fields] OR schizophreniformic[All Fields] OR schizophreniforms[All Fields] OR schizophrenigenesis[All Fields] OR schizophreniics[All Fields] OR schizophrenikern[All Fields] OR schizophrenine[All Fields] OR schizophreniologists[All Fields] OR schizophreniphorm[All Fields] OR schizophrenique[All Fields] OR schizophreniques[All Fields] OR schizophrenis[All Fields] OR schizophrenisation[All Fields] OR schizophrenism[All Fields] OR schizophreniucs[All Fields] OR schizophrenix[All Fields] OR schizophrenix's[All Fields] OR schizophreniz[All Fields] OR schizophrenization[All Fields] OR schizophrenized[All Fields] OR schizophrenjeforschung[All Fields] OR schizophrenlcs[All Fields] OR schizophrenle[All Fields] OR schizophreno[All Fields] OR schizophrenoform[All Fields] OR schizophrenogenesis[All Fields] OR schizophrenogenic[All Fields] OR schizophrenoid[All Fields] OR schizophrenomimetic[All Fields] OR schizophrenomimetics[All Fields] OR schizophrenosimilar[All Fields] OR schizophrens[All Fields] OR schizophrenuc[All Fields] OR schizophreny[All Fields])) OR (schizoaffectieve[All Fields] OR schizoaffectif[All Fields] OR schizoaffectifs[All Fields] OR schizoaffective[All Fields] OR schizoaffective'[All Fields] OR schizoaffectively[All Fields] OR schizoaffectives[All Fields] OR schizoaffectives'[All Fields] OR schizoaffectivity[All Fields])) OR (("bipolar disorder"[MeSH Terms] OR ("bipolar"[All Fields] AND "disorder"[All Fields]) OR "bipolar disorder"[All Fields] OR "manic"[All Fields]) AND ("stupor"[MeSH Terms] OR "stupor"[All Fields]))) NOT comment[Publication Type]) NOT case reports[Publication Type]) NOT letter[Publication Type]) NOT review[Publication Type]
